# Indications and Long-Term Outcomes of Using Mycophenolate Mofetil Monotherapy in Substitution for Calcineurin Inhibitors in Liver Transplantation

**DOI:** 10.3389/ti.2025.13790

**Published:** 2025-02-21

**Authors:** Carlos Jiménez-Romero, Iago Justo Alonso, Oscar Caso Maestro, Alejandro Manrique Municio, Álvaro García-Sesma, Jorge Calvo Pulido, Félix Cambra Molero, Carmelo Loinaz Segurola, Cristina Martín-Arriscado, Anisa Nutu, Alberto Marcacuzco Quinto

**Affiliations:** ^1^ Unit of Hepato-Pancreato-Biliary Surgery and Abdominal Organ Transplantation, Doce de Octubre University Hospital, Madrid, Spain; ^2^ Department of Surgery, Faculty of Medicine, Complutense University, Madrid, Spain; ^3^ Clinical Research Department, Instituto de Investigación (imas12), Madrid, Spain

**Keywords:** mycophenolate mofetil, tacrolimus, cyclosporine, immunosuppression minimization, liver transplantation, chronic kidney dysfunction

## Abstract

Switching the use of calcineurin inhibitors (CNIs), as basal immunosuppression in liver transplantation (LT) patients, for that of mycophenolate mofetil monotherapy (MMF-MT) is currently considered a good measure in recipients with chronic kidney disease (CKD) and other CNI-related adverse effects. We analyzed a retrospective cohort series of 324 LT patients who underwent long-term follow-up and were switched from CNI immunosuppression to MMF-MT due to CKD and other CNI-related adverse effects (diabetes, hypertension, infection). The median time on MMF-MT was 78 months. The indication for MMF-MT was CKD alone or associated with CNI-related adverse effects in 215 patients, diabetes in 61, hypertension in 42, and recurrent cholangitis in 6. Twenty-four (7.4%) patients developed non-resistant acute rejection post-MMF-MT, and 48 (14.8%) patients experienced MMF-related adverse effects, with MMF-MT withdrawn in only 8 (2.5%) patients. In the comparison between the pre-MMF-MT period and the last outpatient review, using a repeated measures model and taking each patient as its own comparator, we demonstrated a significant increase in GFR and significant decrease in creatinine and ALT values, remaining the other variables (diabetes, hypertension, and hematological and AST) within similar levels. Five-year survival post-MMF-MT conversion was 75.3%. MMF-MT significantly improved renal function, was well tolerated, and had a low rejection rate.

## Graphical Abstract

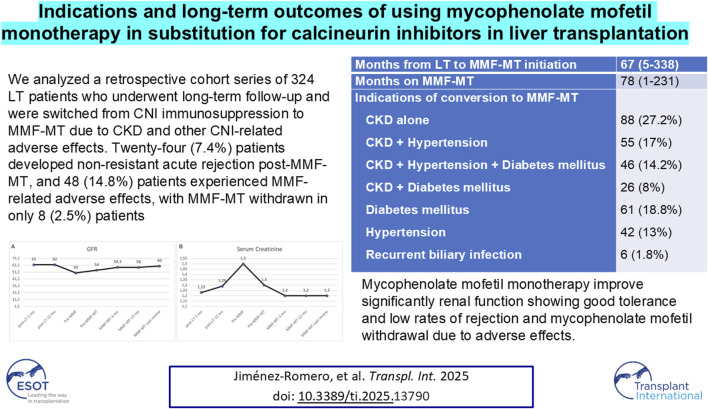

## Introduction

Currently, calcineurin inhibitors (CNIs) are the standard therapy for maintenance immunosuppression in patients who undergo liver transplantation (LT), with a preference for tacrolimus over cyclosporine [1]. However, CNI drugs are often associated with several adverse effects, such as: nephrotoxicity, chronic kidney disease (CKD), neurotoxicity, diabetes, arterial hypertension, cardiovascular complications, hyperlipidemia, hyperuricemia, hepatocellular carcinoma (HCC) recurrence, *de novo* malignancies and infections [2–7]. The use of tacrolimus is associated with improved renal function than the use of CyA [4], especially when a low dose of tacrolimus is combined with mycophenolate mofetil (MMF) [8–10]. Conversely, some immunosuppressive changes have been introduced to prevent or reduce the adverse effects related to CNIs, such as the minimization or substitution of CNIs for either MMF monotherapy (MT) or the mTORi-MT regimen [11–13]. The term “immunosuppression minimization” is defined as the lowest dose of immunosuppressive drugs compatible with a rejection-free state and the absence of clinically adverse effects [14, 15].

Mycophenolic acid (MPA) is the pharmacokinetically active product of MMF with potent inhibitory effects on *de novo* purine synthesis and T and B lymphocyte proliferation. Nevertheless, several adverse effects have also been associated with the use of MMF, such as myelotoxicity (anemia, leukopenia, and thrombocytopenia) and gastrointestinal symptoms (diarrhea, nausea, vomiting, abdominal pain, hemorrhage) [16–19], as well as teratogenicity [20]. Despite these drawbacks, switching from CNIs to MMF-MT is currently considered a good measure to improve kidney function in patients who develop post-LT CKD [16, 18, 19, 21–24], hypertension [16, 21, 22, 25], and diabetes mellitus [17, 22]. The presence of hypertension, with a prevalence of approximately 70% in LT patients, increases the risk of chronic kidney disease (CKD) and cardiovascular disease development and is associated with a higher mortality risk more than 1 year after LT [26].

The aim of this retrospective study is to describe our experience switching CNI immunosuppression to that with MMF-MT in LT patients who develop CNI-related adverse effects throughout a long-term follow-up. To our knowledge, this study is the largest single-center study reported using MMF-MT in LT patients.

## Patients and Methods

### Population and Study Design

Between April 1986 and June 2022, we performed a total of 2,204 LTs at our institution. For this study, we recorded the data of 324 LT recipients among a total of 1,697 who underwent LT between January 1997 (the first patient included in MMF-MT) and June 2022 and were subsequently converted from immunosuppression with combined CNI-MT or CNI + MMF to MMF-MT. In this retrospective single-center cohort study, we analyzed the impact of MMF-MT on toxicity or adverse effects (CKD, hypertension, diabetes mellitus, and recurrent biliary infection) in patients previously undergoing immunosuppression with CNIs and the incidence of rejection and adverse effects related to the use of MMF-MT.

The inclusion criteria for conversion from CNI-MT to MMF-MT were as follows: patients >18 years usually with more than 2 years of follow-up after LT, stable liver graft function and the absence of acute rejection in the last year before MMF-MT conversion. This study was closed on December 2023, and all patients were followed for at least 1.5 years after conversion to MMF-MT. Medical history of liver retransplantation, previous renal transplant, and hepatocellular carcinoma recurrence were considered exclusionary for this study. Informed consent for MMF-MT was obtained from all patients included in this study.

This research was performed in accordance with the ethical guidelines of the 1964 Helsinki Declaration and its later amendments and was approved by our Institutional Review Board (Research Registry no 24/025). The need for local clinical research ethics committee approval was waived because of the retrospective nature of the research.

### Baseline Data

The following patient data were retrospectively collected: age, sex, body mass index (BMI), Child‒Pugh and MELD scores, presence of arterial hypertension or diabetes, LT indications, pre-LT recipient laboratory values, type of donors and graft steatosis, type of CNI, rate and grade of post-LT acute rejection, median time elapsed from LT to initiation of MMF-MT and median time on MMF-MT, indications of conversion to MMF-MT, rate of post-MMF-MT acute rejection and therapy, adverse effects related to MMF-MT, causes of MMF-MT withdrawal, and the need for dialysis and kidney transplant during the follow-up of MMF-MT patients.

Determinations were taken of post-MMF-MT laboratory parameters (serum glucose, hematological, kidney and liver function values), doses of MMF and blood levels of MPA throughout the follow-up at different periods [pre-MMF-MT (combined CNI-MMF), 3, 6, and 12 months post-MMF-MT, and at the end of the study]. Comparisons between the variables (diabetes, hypertension, and hematological, kidney and liver function) of pre-MMF-MT patients and the final outpatient review of MMF-MT patients were performed. The sample was divided into two eras (first era: 1999–2011; second era: 2012–2023), that were compared regarding acute rejection, adverse effects, causes MMF-MT withdrawal and patient survival was performed between both groups of patients.

### Variable Definitions

CKD was defined as a GFR <60 mL/min/1.73 m^2^ or markers of kidney damage, or both, of at least 3 months in duration, and estimation of the GFR was performed according to the CKD-EPI equation [27]. Arterial hypertension was defined as a systolic blood pressure >140 mmHg and/or a diastolic blood pressure >90 mmHg on three consecutive measurements within three to 6 months [28]. A diagnosis of diabetes mellitus was established if fasting plasma glucose was ≥126 mg/dL or if 2 h plasma glucose levels were ≥200 mg/dL, according to the ADA criteria [29]. Anemia was defined as hemoglobin <8 g/dL; leukopenia was defined as a white blood cell count <2,500/mm^3^; and thrombocytopenia was defined as a platelet count <60,000/mm^3^. The diagnosis of acute rejection was performed by liver graft biopsy or empirically by alteration of liver function tests. Acute rejection was classified according to the Banff grades [30].

### Immunosuppression

The initial immunosuppressive regimen comprised CNI (cyclosporine or tacrolimus) and steroids with or without MMF. Steroids were discontinued between 3 and 6 months post-transplantation. The dose of tacrolimus was adjusted to achieve target blood trough levels of 10–15 ng/mL for the first month, 7–9 ng/mL within the first year, 5 ng/mL between the 2nd and 4th years, and between 4 and 5 ng/mL thereafter. The dose of oral cyclosporine was adjusted to maintain blood trough levels between 200 and 300 ng/mL for the first month and between 150 and 200 thereafter.

In the presence of severe adverse effects associated with CNIs, such as renal dysfunction, diabetes, and hypertension, MMF was introduced to reduce CNI levels by half. Conversion from CNI to MMF-MT was performed on long-term follow-up recipients with stable liver function, starting at a dose of 500 mg of MMF twice daily, which was subsequently increased up to 1 g twice daily, followed by a gradual reduction in CNI until complete withdrawal. For patients on MMF-MT, MMF was administered at a dose capable of maintaining MPA levels between 2–4 ng/mL. Currently, the period from the introduction of MMF to CNI withdrawal is between 1 and 2 months, with posterior review in the outpatient clinic at 15, 30, and 90 days and routine follow-up every 6 months thereafter.

Patients on MMF-MT who showed liver dysfunction or biopsy-proven acute rejection grade I/II were initially treated with increasing doses of MMF (up to 1 g/12 h) to achieve MPA levels between 2–4 ng/mL or with 0.5–1 g of methylprednisolone intravenously for 3 days. Tacrolimus, cyclosporine or mTORi were reintroduced in cases of resistant acute rejection. In the presence of moderate-severe adverse effects, MMF was reduced or withdrawn and CNI was reintroduced. The dose of MMF was adjusted according to the protocol based on blood levels and liver function.

### Statistical Analysis

Qualitative variables are expressed as absolute numbers, and relative frequencies are expressed as percentages. Associations were analyzed via the chi-square test or Fisher’s exact test, when applicable. Most quantitative variables did not have a normal distribution according to the Kolmogorov–Smirnov test; therefore, all the quantitative variables are expressed as medians and percentiles and are expressed between 0 and 100. The relationships between quantitative variables were analyzed via the Mann–Whitney U test. A repeated measures model was used, taking each patient as its own comparator, in order to evaluate different key parameters pre and post MMF-MT. Survival analysis was performed via the Kaplan–Meier estimator and the log-rank test. A *p* value of <0.05 was considered to indicate statistical significance. Statistical analysis was performed via SPSS Statistics, version 25 (SPSS, Inc., Chicago, IL, United States).

## Results

### Recipient and Donor Characteristics

From January 1997 to June 2022, a total of 1,697 patients underwent LT and were immunosuppressed with CNI. A group of 324 patients was initially treated with CNI standard immunosuppression (252 with tacrolimus-based and 72 with cyclosporine-based). The median recipient age was 55 (19–70) years, and the median MELD score was 15 (6–35). Alcoholic cirrhosis, hepatitis C virus (HCV) cirrhosis and HCC were the most frequent indications for LT.

Concerning pre-LT laboratory variables, the median serum creatinine value was 1.1 (0.5–1.9) mg/dL, and the median GFR was 70.2 (36–111) mL/min/1.73 m^2^. Livers from donation after brain death (DBD) were used in 289 (89.2%) patients, and livers from donors with uncontrolled circulatory death (uDCD) were used in 16 (4.9%) patients. The remaining characteristics of the recipients and donors are detailed in Table 1.

**TABLE 1:** Characteristics of recipients and donors converted from CNI to MMF-MT.

Pre-LT variables	n = 324
Age (yr)	55 (19–70)
Sex (M/F)	242/82 (74.7%/25.3%)
Body mass index	26.9 (14.5–46)
Child‒Pugh score A B C	32 (9.9%) 166 (51.2%) 126 (38.9%)
MELD score	15 (6–35)
Hypertension	50 (15.4%)
Diabetes mellitus	79 (24.4%)
LT indications	
Alcoholic cirrhosis	150 (46.3%)
Hepatitis C virus cirrhosis	133 (41%)
Hepatocellular carcinoma	81 (25%)
Hepatitis virus B cirrhosis	43 (13.3%)
Pre-LT laboratory values of recipients	
Serum creatinine (mg/dL)	1.1 (0.5–1.9)
GFR (mL/min/1.73 m^2^)	70.2 (36–111)
Serum glucose (mg/dL)	109 (81–290)
Bilirubin (mg/dL)	2.15 (0.9–41)
Na (mEq/L)	135 (128–144)
K (mEq/L)	4.3 (3–5)
Cholesterol (mg/dL)	136 (83–274)
Leukocytes/mm^3^	5,100 (2,100–16,000)
Hemoglobin (g/dL)	12.6 (10–15.1)
Platelets/mm^3^ × 10^3^	73 (20–243)
Type of donors	
Donation after brain death	289 (89.2%)
Donation after circulatory death	16 (4.9%)
Split-liver	9 (2.8%)
Living donor	7 (2.2%)
Pediatric	3 (0.9%)
Steatosis No Microsteatosis Macrosteatosis N/A	61 (18.8%) 75 (23.1%) 138 (42.6%) 50 (15.4%)
Grade of macrosteatosis Mild (<30%) Moderate (30%–60%) Severe (>60%)	101 (31.1%) 35 (10.8%) 2 (0.6%)

GFR, glomerular filtration rate; MELD, model for end-stage liver disease.

### Pre- and Post-MMF-MT Characteristics

Immediately after LT, tacrolimus-based immunosuppression was used in 252 (77.8%) patients vs. the 72 (22.2%) who received cyclosporine-based immunosuppression. One episode of acute rejection after LT occurred in 90 (27.8%) patients, and two or more episodes in 23 (7.1%), with rejection grades I-II appearing in 106 (32.7%) patients.

The median time from LT to the initiation of MMF-MT was 67 (5–338) months, whereas the median time from the initiation of the combined MMF-CNI or CNI-alone regimen to CNI withdrawal and switch to MMF-MT was 18 (0–170) months. The overall median follow-up time of patients on MMF-MT was 68 (1–231) months.

The indication for switching from CNI to MMF-MT was CKD in 215 (66.4%) patients (CKD on its own in 88 patients and associated with hypertension in 55 patients, diabetes and hypertension in 46 patients, and associated with diabetes in 26 patients), diabetes mellitus on its own in 61 (18.8%) patients, hypertension in 42 (13%) patients, and recurrent biliary infection in 6 (1.8%) patients.

Just before shifting from tacrolimus to MMF-MT, 228 (70.3%) patients were on tacrolimus immunosuppression (associated with MMF in 220 patients and monotherapy in 8 patients), and 96 patients were on cyclosporine immunosuppression (associated with MMF in 91 patients and monotherapy in 5 patients).

Twenty-four (7.4%) patients experienced acute rejection after conversion from CNI to MMT-MT; 14 (4.3%) patients were diagnosed by liver biopsy (grade I/II in 13), and 10 (3.1%) were empirically diagnosed by liver dysfunction. All patients responded completely to rejection therapy (steroids and reintroduction of tacrolimus [20 patients], cyclosporine [1 patient], or mTORi [3 patients]). Forty-eight (14.8%) patients developed adverse effects related to MMF-MT, with the most common diarrhea (5.6%), vomiting (1.9%), and leukopenia (5.9%).

MMF-MT withdrawal was performed in 42 (12.9%) patients, due to *de novo* tumors in 13 (4%) patients (substitution of MMF-MT by mTORi monotherapy), biopsy-proven rejection in 8 (2.5%), liver dysfunction in 6 (1.8%), liver retransplantation in 4 (1.2%), kidney retransplantation in 3 (0.9%) and adverse effects in 8 (2.5%). With respect to side effects, MMF-MT withdrawal was performed in 5 (1.5%) patients with persistent chronic diarrhea despite a change from MMF to mycophenolate sodium salt and in 3 (0.8%) patients with leukopenia (Table 2). The remaining patients with adverse effects improved with a reduction in MMF dosage.

**TABLE 2:** Pre- and post-MMF-MT rejection, adverse effects and MMF withdrawal.

Post-LT CNI immunosuppression Tacrolimus-based Cyclosporine-based	252 (77.8%) 72 (22.2%)
Post-LT acute rejection	113 (34.9%)
Number of episodes 1 ≥2	90 (27.8%) 23 (7.1%)
Grade of rejection I II III	47 (14.5%) 59 (18.2%) 7 (2.1%)
Months from LT to MMF-MT initiation	67 (5–338)
Months from MMF initiation to MMF-MT	18 (0–170)
Months on MMF-MT (last outpatient review)	78 (1–231)
Indications of conversion from CNI to MMF-MT CKD alone CKD + Hypertension CKD + Hypertension + Diabetes mellitus CKD + Diabetes mellitus Diabetes mellitus Hypertension Recurrent biliary infection	88 (27.2%) 55 (17%) 46 (14.2%) 26 (8%) 61 (18.8%) 42 (13%) 6 (1.8%)
Pre-MMF-MT (CNI immunosuppression) Tacrolimus + MMF Tacrolimus Cyclosporine + MMF Cyclosporine	220 (67.9%) 8 (2.5%) 91 (27.8%) 5 (1.5%)
Post-MMF-MT acute rejection	24 (7.4%)
Diagnosis by liver biopsy Grade I Grade II Grade III Diagnosis by liver dysfunction	14 (4.3%) 8 (2.5%) 5 (1.5%) 1 (0.3%) 10 (3.1%)
Acute rejection therapy Tacrolimus Cyclosporine mTORi	20 (6.2%) 1 (0.3%) 3 (0.9%)
Adverse effects Diarrhea Vomiting Leukopenia Anemia Asthenia	48 (14.8%) 18 (5.6%) 6 (1.9%) 19 (5.9%) 3 (0.9%) 2 (0.6%)
Causes of MMF-MT withdrawal De novo tumors Rejection Liver dysfunction (no liver biopsy) Liver retransplantation Kidney transplantation Adverse effects Diarrhea Leukopenia	42 (12.9%) 13 (4%) 8 (2.5%) 6 (1.8%) 4 (1.2%) 3 (0.9%) 8 (2.5%) 5 (1.5%) 3 (0.9%)

CKD, chronic kidney disease; CNI, calcineurin inhibitor; LT, liver transplantation; MMF-MT, mycophenolate mofetil monotherapy.

### Dosage of MMF and Monitoring of MPA Levels Through Follow-Up

The dosage of MMF was adjusted according to MPA plasma levels, resulting in great variability among patients. The overall daily dose of MMF and median MPA plasma levels in patients on combined CNI-MMF therapy (just before conversion to MMF-MT) and in MMF-MT patients after 3, 6, and 12 months and at the last outpatient review (median period of 78 months) are detailed in Table 3, where it can be observed that MPA median plasma levels were similar in the pre-MMF-MT period or combined CNI-MMF [2.6 (0.1–15 ng/dL)] and at the last outpatient review [2.7 (0.2–15) ng/dL; *p* = 0.527].

**TABLE 3:** Overall daily doses of MMF and monitoring of MPA levels during follow-up.

MMF dose (mg/d)	Pre-MMF-MT (CNI-MMF)	MMF-MT (3-mo)	MMF-MT (6-mo)	MMF-MT (12-mo)	^a^MMF-MT (median: 78-mo) (last outpatient review)
500	8 (2.5%)	4 (1.2%)	2 (0.6%)	7 (2.2%)	7 (2.2%)
750	3 (0.9%)	4 (1.2%)	4 (1.2%)	8 (2.5%)	18 (5.6%)
1,000	101 (31.2%)	54 (16.7%)	47 (14.5%)	51 (15.7%)	110 (33.9%)
1,250	4 (1.2%)	15 (4.6%)	13 (4%)	20 (6.2%)	15 (4.6%)
1,500	68 (20.9%)	89 (27.5%)	78 (24.1%)	86 (26.5%)	60 (18.5%)
1,750	3 (0.9%)	11 (3.4%)	13 (4%)	12 (3.7%)	4 (1.2%)
2,000	100 (30.9%)	130 (40.1%)	139 (42.9%)	109 (33.6%)	99 (30.6%)
N/A	37 (11.4%)	17 (5.2%)	28 (8.6%)	31 (9.6%)	11 (3.4%)
MPA median levels (ng/mL)	2.6 (0.1–15)	3.3 (0.4–13)	3.3 (0.5–12)	3 (0.5–19)	2.7 (0.2–15)

^a^ Comparison between MPA levels in the pre-MMF-MT period and the last outpatient visit (*p* = 0.527). CNI, calcineurin inhibitor; MMF-MT, mycophenolate mofetil monotherapy; MPA, mycophenolic acid.

### Comparison of Characteristics Between Pre-MMF-MT and Final Review

**FIGURE 1:**
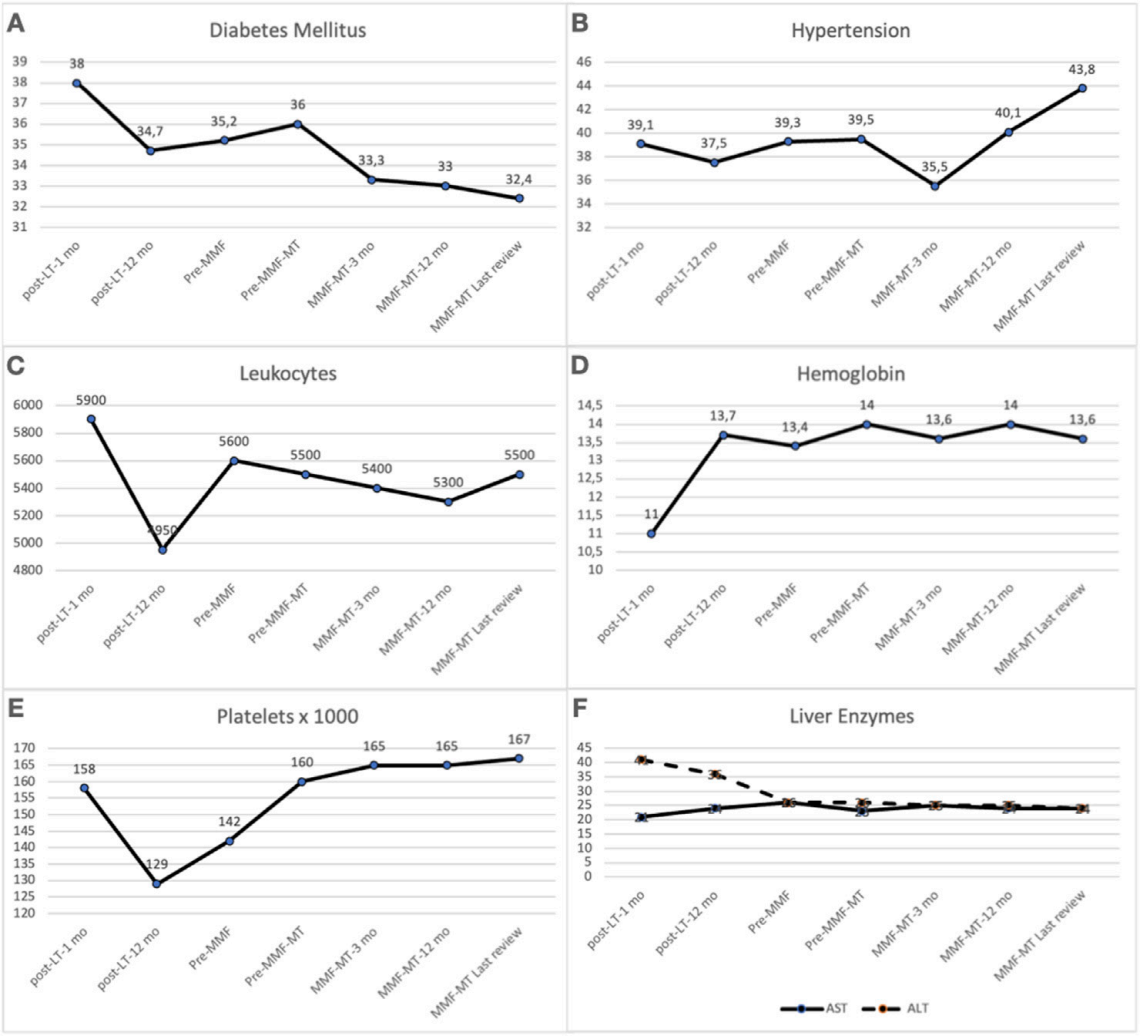
Comparison between the pre-MMF-MT period (combined MMF-CNI or CNI alone) and the final period of MMF-MT (last outpatient review) regarding the frequency of diabetes mellitus **(A)** (*P* = 0.603) and hypertension **(B)** (*P* = 0.141) and the median values of leukocytes **(C)** (*P* = 0.391), hemoglobin **(D)** (*P* = 0.115), platelets **(E)** (*P* = 0.210), AST **(F)** (*P* = 0.471) and ALT **(F)** (*P* = 0.106). *ALT, alanine amino transferase; AST, aspartate amino transferase; CNI, calcineurin inhibitor; GFR, glomerular filtration rate; MMF-MT, mycophenolate mofetil monotherapy.

**FIGURE 2:**
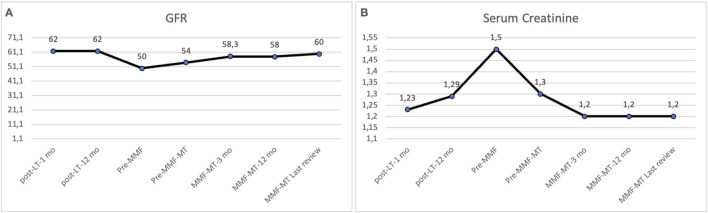
Comparison of renal function between the pre-MMF-MT period (combined MMF-CNI or CNI alone) and the final period of MMF-MT (last outpatient review). The median values of the GFR **(A)** increased significantly at the end of the study (56.5 mL/min/1.73 m^2^ vs. 61 mL/min/1.73 m^2^; *p* = 0.001). The median serum creatinine **(B)** value decreased at the end of the study, but the difference was not statistically significant (*p* = 0.112). *CNI, calcineurin inhibitor; GFR, glomerular filtration rate; MMF-MT, mycophenolate mofetil monotherapy.

The frequency of diabetes and hypertension and laboratory values of hematological, renal and liver function during long-term follow-up are shown in Table 4. In the comparison of the median values of variables between the pre-MMF-MT period (combined MMF-CNI or CNI alone) and the last outpatient review on MMF-MT (median of 78 months), only the median GFR value was significantly greater in the last review on MMF-MT [56 (15–126) mL/min/1.73 m^2^ vs. 61 (7–134) mL/min/1.73 m^2^; *p* = 0.001], whereas the frequency of diabetes and hypertension and laboratory values of hematological variables, serum creatinine and liver function (AST and ALT) did not show significant differences between the two periods (Figures 1, 2). The 6 patients who were switched to MMF-MT due to biliary infection did not experience any new episodes of recurrent biliary cholangitis. On the other hand, using a repeated measures model, taking each patient as its own comparator, we found a statistically significant increase in GFR, statistically significant decrease in creatinine and statistically lower value of ALT at the last outpatient review in comparison with the pre-MMF-MT period (combined MMF-CNI or CNI alone) (Table 4).

**TABLE 4:** Comparison of variables between the pre-MMF-MT period and last review^a^.

Diabetes mellitus	OR	p	CI 95%
Pre-MMF-MT	1.387	0.569	0.450 to 4.276
Last review	1.869	0.117	0.525 to 15.537
Hypertension			
Pre-MMF-MT	0.429	0.179	0.395 to 1.249
Last review	0.376	0.416	0.239 to 2.723
Leukocytesx 10^3^			
Pre-MMF-MT	−140	0.695	−842 to 5,617
Last review	310	0.389	399 to 1,016
Hemoglobin			
Pre-MMF-MT	0.299	0.347	−0.032 to 0.923
Last review	0.013	0.967	0.613 to 639
Platelets			
Pre-MMF-MT	492	0.938	0.118 to 12.836
Last review	467	0.460	0.772 to 17.071
AST			
Pre-MMF-MT	−4.015	0.084	8.575 to 0.545
Last review	−4.077	0.080	8.648 to 0.494
ALT			
Pre-MMF-MT	−8.817	0.001	−14.243 to 3.391
Last review	−12.295	0.000	−17.734 to 6.855
GFR			
Pre-MMF-MT	4.223	0.000	2.233 to 6.213
Last review	6.920	0.000	4.924 to 8.917
Creatinine			
Pre-MMF-MT	−0.418	0.009	−0.733 to −0.104
Last review	−0.375	0.020	−0.691 to −0.059

ALT, alanine amino transferase; AST, aspartate amino transferase; GFR, glomerular filtration rate; MMF-MT, mycophenolate mofetil monotherapy.

^a^ Comparison between pre-MMF-MT and last review using a repeated measures model taking each patient as its own comparator.

Concerning comparison between both eras, we observed a significantly higher incidence of hypertension (*p* = 0.019) and diabetes mellitus (*p* = 0.023) before LT in the first era, and a significantly higher incidence of HCC (*p* < 0.001) in the second era, showing significant differences (*p* < 0.001) between the eras regarding indications of conversion to MMF-MT (Table 5).

**TABLE 5:** Results according to eras of LT recipients converted from CNI to MMF-MT.

	First Era (1999–2011) (n = 161)	Second Era (2012–2023) (n = 163)	*P*
Age (yr)	54 (22–70)	56 (19–70)	
Sex (M/F)	111(68.9%)/50 (31.1%)	131 (80.4%)/32 (19.6%)	0.018
Hypertension	33 (20.5%)	17 (10.4%)	0.019
Diabetes mellitus	32 (19.9%)	47 (28.8%)	0.023
MELD Score	15 (7–23)	15 (6–35)	
LT indications			
Alcoholic cirrhosis	67 (41.6%)	83 (50.9%)	0.093
Hepatitis C virus cirrhosis	63 (39.1%)	70 (42.9%)	0.485
Hepatocellular carcinoma	24 (14.9%)	57 (35.4%)	<0.001
Hepatitis virus B cirrhosis	24 (15%)	19 (11.7%)	0.388
Steatosis No Microsteatosis Macrosteatosis N/A	24 (14.9%) 42 (26.1%) 71 (44.1%) 24 (14.9%)	37 (22.7%) 33 (20.2%) 67 (41.1%) 26 (15.9%)	0.062
Indications of conversion from CNI to MMF-MT CKD alone CKD + Hypertension CKD + Hypertension + Diabetes mellitus CKD + Diabetes mellitus Diabetes mellitus Hypertension Recurrent biliary infection	42 (26.1%) 18 (11.2%) 30 (18.6%) 10 (6.2%) 26 (16.1%) 31 (19.2%) 4 (2.5%)	46 (28.2%) 37 (22.7%) 16 (9.8%) 16 (9.8%) 35 (21.5%) 11 (6.7%) 2 (1.2%)	<0.001
Post-MMF-MT acute rejection Diagnosis by liver biopsy Grade I Grade II Grade III Diagnosis by liver dysfunction	11 (6.8%) 6 (3.7%) 3 (1.8%) 2 (1.2%) 1 (0.6%) 5 (3.1%)	13 (7.9%) 8 (4.9%) 5 (3.1%) 3 (1.8%) 0 (0%) 5 (3.1%)	0.694
Adverse effects Diarrhea Vomiting Leukopenia Anemia Asthenia	26 (16.1%) 7 (4.3%) 4 (2.5%) 13 (8.1%) 1 (0.6%) 1 (0.6%)	22 (13.5%) 11 (6.7%) 2 (1.2%) 6 (3.7%) 2 (1.2%) 1 (0.6%)	0.667
Causes of MMF-MT withdrawal *De novo* tumors Rejection Liver dysfunction (no liver biopsy) Liver retransplantation Kidney transplantation Adverse effects • Diarrhea • Leukopenia	26 (16.1%) 11 (6.7%) 4 (2.5%) 3 (1.9%) 2 (1.2%) 2 (1.2%) 4 (2.5%) 3 (1.9%) 1 (0.6%)	16 (9.8%) 2 (1.2%) 4 (2.4%) 3 (1.8%) 2 (1.2%) 1 (0.6%) 4 (2.4%) 1 (0.6%) 3 (1.8%)	0.460
Actuarial patient survival after MMF-MT 1-y 3-y 5-y 10-y	93.8% 82.3% 70.1% 51.9%	97.9% 91.8% 80.9% 64.3%	0.089

**FIGURE 3:**
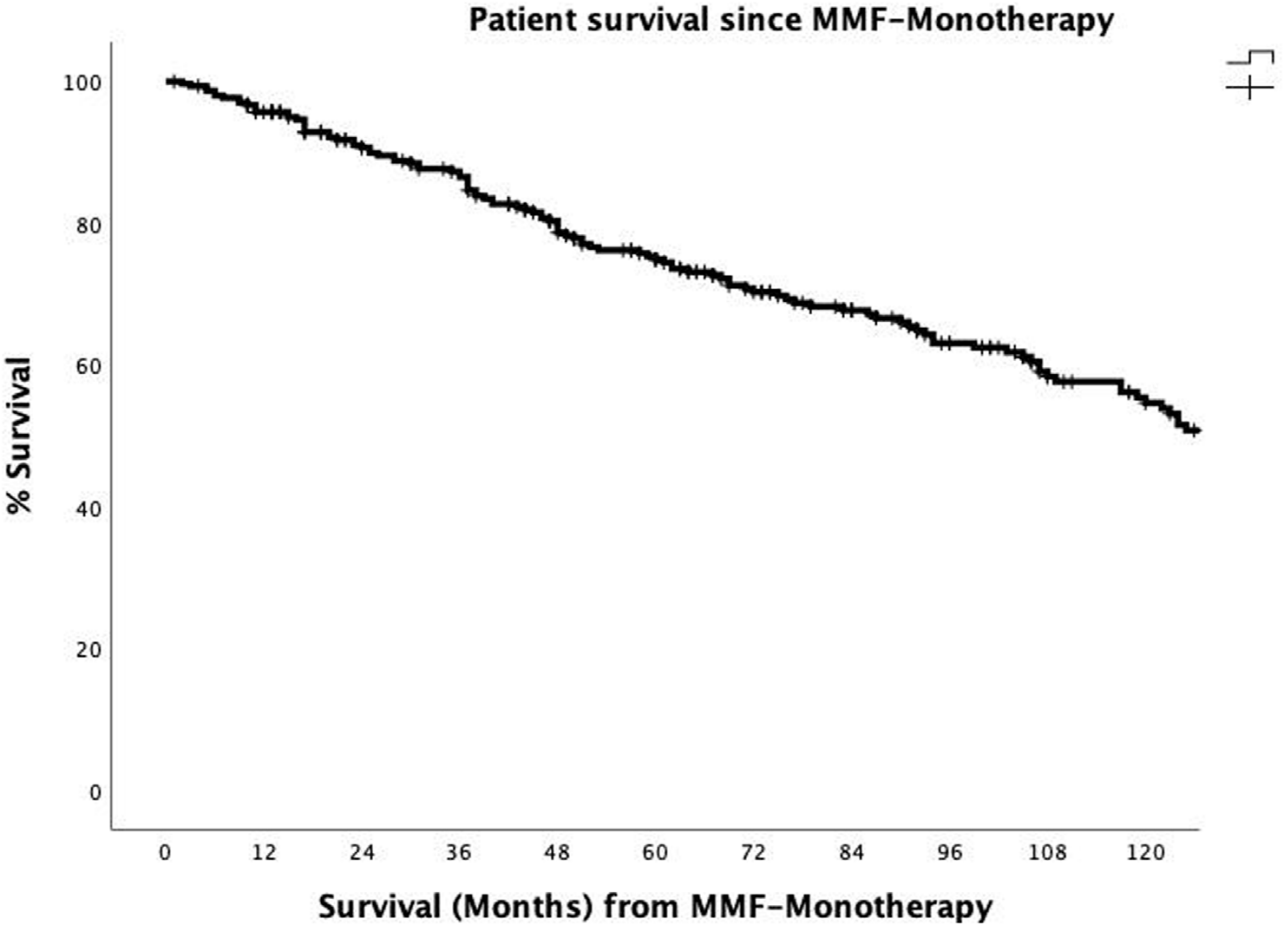
The actuarial patient survival rates after conversion from CNI immunosuppression to MMF-MT (mycophenolate mofetil monotherapy) at 1, 3, 5, and 10 years were 95.7%, 86.5, 75.3%, and 54.6%.

The overall actuarial patient survival rates at 1, 3, 5, and 10 years after the onset of MMF-MT were 95.7%, 86.5%, 75.3%, and 54.6%, respectively (Figure 3). The actuarial patient survival rate at 1, 3, 5, and 10 years after the onset of MMF-MT were 93.8%, 82.3%, 70.1%, and 51.9%, respectively, in era 1, whereas in the second era patient survival rate was 97.9%, 91.8%, 80.9%, and 64.3%, respectively. (*p* = 0.089) (Table 5).

## Discussion

The use of CNIs within the first 12 months after LT is a risk factor for renal failure [31], with a cumulative incidence of advanced CKD (GFR ≤29 mL/min) of 8% at 1 year and 18.1% at 5 years [32]. Renal function should improve more notably when the CNI is completely withdrawn than when it is partially withdrawn [33–35]. The substitution of CNI drugs for MMF-MT has been indicated mainly to halt or improve CKD and other CNI-induced adverse effects [16, 18, 19, 21–23, 33–35]. Other less frequent indications for shifting to MMF-MT were neurological or cardiovascular complications, risk of tumor recurrence [19], and metabolic disorders [18]. The most frequent indications for LT in our series were alcoholic cirrhosis, HCV, hepatitis B virus (HBV) and HCC, and tacrolimus was the most commonly used immunosuppressor, with an overall rate of acute rejection of 34.6% after LT. As in other reported studies [2–7], the main reasons for conversion from CNIs to MMF-MT in our study were the presence of CKD on its own or in association with diabetes or arterial hypertension and other adverse effects related to the use of CNIs, such as the presence of isolated diabetes, hypertension, or recurrent biliary infection.

Some researchers are reluctant to switch CNIs for MMF-MT in patients with previous history of graft rejection using CNIs [34], but the usual practice of other researchers is to switch CNI for MMF-MT in patients in the absence of acute rejection for 6–15 months before conversion [16, 17, 19, 36], the presence of stable liver function [16, 17, 21], and the absence of anemia, leukopenia and thrombocytopenia [36].

According to several studies, the time elapsed from LT to the onset of MMF-MT was between 27–81 months [16, 17, 19, 21, 22, 37–39], whereas in our experience, it corresponded to a median period of 72 months. The period of conversion from MMF-CNI to CNI withdrawal and the initiation of MMF-MT has been reported to last from 2 weeks to 9 months [16–19, 21, 25, 37, 40]; to 19 months in our experience. However, following our long-term experience, our period from the introduction of MMF to complete CNI withdrawal has been reduced to 1–2 months, with routine follow-up at 15, 30, and 90 days to detect occasional acute rejection or adverse effects.

The mean follow-up period of several studies of patients on MMF-MT is between 12 and 48 months [16–19, 21, 22, 38, 39], whereas our median follow-up time for patients on MMF-MT has reached 78 months.

Conversion from CNIs to MMF-MT is usually performed with an initial dose of 500 mg/12 h, reaching a dose of 1 g/12 h for 2–4 weeks simultaneously with a gradual reduction in CNI, usually by 25% at a time, until complete withdrawal [16, 17, 23, 25, 35]. Several authors have advised on maintaining MPA plasma levels between 1 and 3.5 ng/mL [41] or between 2 and 4 ng/mL, adjusting the MMF dosage according to the degree of renal dysfunction and monitoring of MPA plasma levels [21, 35, 39, 42, 43] because impaired renal function is correlated with a decrease in the clearance of MPA metabolites, which consequently increases the plasma concentration of MPA metabolites and augments immunosuppression [44]. Although MMF-MT may be a risk factor for liver rejection [45], we agree with other experiences that monitoring MPA levels can improve the management of immunosuppression [46] and may even limit the risk of rejection or drug toxicity [19, 23, 38, 45]. Thus, with MPA level monitoring, we adjusted the MMF doses between 500 and 2,000 mg/d to obtain median MPA levels between 2.5 and 3.3 ng/mL, maintaining 135 (38.6%) patients without rejection on a dose ≤1,000 mg/d of MMF at the last outpatient review. The monitoring of MPA levels allows the minimization of immunosuppression and prevents MMF-related adverse effects. However, AUC is considered the goal standard for measuring MPA levels [47], although in our experience it has been very impractical owing to the time and number of determinations it requires, especially among our high volume of patients, many of whom do not live in our city. Therefore, due to its simplicity and the need for only one determination per visit, it was decided in our center to use MPA levels.

Due to the mentioned risk of acute rejection, it is advisable to not attempt MMF-MT in poor MMF absorbers, which have been defined as patients with MPA levels <0.5 ng/mL after daily intake of ≥1,000 mg/d MMF or MPA levels <1 ng/mL after daily intake of 1,500 mg/d [48].

Substitution of CNIs by MMF-MMF has been associated with a rate of acute rejection ranging from 4% to 21.4% [16–19, 21, 22, 25, 37, 38, 48, 49]. Our rate of acute rejection was 7.4%, and as other researchers [23] have reported, diagnosis was performed either by liver biopsy or empirically by alteration of liver function. All our patients with acute rejection responded successfully with steroids and/or reintroduction of CNI or mTORi monotherapy, and no patients developed chronic rejection. However, late acute liver rejection can occur, and close follow-up during the first year after MMF-MT is advised, as has been previously reported [33].

The development of adverse effects is frequently related to MPA plasma levels higher than 4 ng/mL [41]. There is great variability in MMF-MT-induced side effects, ranging from an incidence between 4.3% and 57% [16, 17, 19, 21, 25, 37], although they are usually controlled by a reduction in the MMF dose [16, 18, 21, 35, 43], with the need for MMF-MT withdrawal in only 2%–11.8% of patients who show gastrointestinal symptoms, pancytopenia or pruritus [17, 19, 22, 25, 39]. In addition, gastrointestinal adverse effects can be improved by switching from MMF to enteric-coated mycophenolate sodium [50, 51]. In our study, 48 (14.8%) patients developed MMF-MT-induced adverse effects, but conversion to CNI was only performed in 8 (2.5%) patients due to failure to control severe diarrhea with enteric-coated mycophenolate sodium or leukopenia. The remaining adverse effects of patients improved when the dose of MMF decreased without the need to return to CNIs. Other reasons for MMT-MT withdrawal in our experience were the presence of *de novo* tumors (13 patients), biopsy-proven rejection or liver dysfunction (14 patients), liver retransplantation (4 patients) and kidney transplantation (3 patients).

Serum creatinine levels improved significantly in between 78.6% and 89% of the patients who were converted from CNIs to MMF-MT [16, 21, 25]. Similarly, replacement of CNIs with MMF-MT significantly increased the mean value of the GFR in patients with CKD [18, 19, 21, 22]. In our study, the comparison between the median values of the GFR in the pre-MMF-MT (CNI therapy) period and the last outpatient control, with a median value of 78 months between the two periods, revealed a significantly greater value of the GFR in the last control (56.5 vs. 61 mL/min/1.73 m^2^). The median serum creatinine value also improved at the last outpatient visit, but the difference was not statistically significant. However, when a repeated measures model was used taking each patient as its own comparator a significant increase of GFR and a significant decrease of serum creatinine and ALT values were demonstrated not showing significant differences regarding the rates of diabetes, hypertension, and values of hematological variables and AST.

In addition, the rates of diabetes mellitus and hypertension and the median values of hematological parameters (hemoglobin, leukocytes and platelets), serum glucose and liver transaminases did not significantly differ between the two periods. Notably, the 6 patients who were converted to MMF-MT due to recurrent cholangitis did not experience any more infection episodes after conversion. Five-year patient survival after conversion from CNI to MMF-MT was reported to be between 70% and 90% in 3 studies [19, 23, 35], and our 5-year patient survival rate was 75.3%. In the comparison of the results after conversion to MMF-MT in both eras we did not find significant differences regarding to the rates of acute rejection, adverse effects and causes of MMF-MT withdrawal, finding a higher patient survival rate in the second era, although statistically unsignificant.

This study has several limitations, such as its retrospective nature, long duration, and single institution design; consequently, it is subject to bias. Future multicenter prospective randomized studies with large samples are necessary to confirm our results.

In conclusion, MMF-MT can be safely used in LT patients with CNI-related adverse effects, such as CKD, hypertension, diabetes and biliary infection. Monitoring of MPA levels allows the reduction of the MMF dose and its adverse effects. The acute rejection rate was low, with a good response to CNI reintroduction or mTORi therapy, and the GFR, creatinine and ALT transaminase improved significantly through long-term follow-up. Comparison of the results between 2 eras did not show significant differences. A good tolerance of MMF-MT and a low rate of MMF-MT withdrawal have been shown.

## Data Availability

The original contributions presented in the study are included in the article/Supplementary Material, further inquiries can be directed to the corresponding author.

## Abbreviations

ALT, alanine amino transferase; AST, aspartate amino transferase; BMI, body mass index; CKD, chronic kidney disease; CNI, calcineurin inhibitor; DBD, donation after brain death; GFR, glomerular filtration rate; HBV, hepatitis B virus; HCC, hepatocellular carcinoma; HCV, hepatitis C virus; LT, liver transplantation; MELD, model for end-stage liver disease; MMF, mycophenolate mofetil; MT, monotherapy; MPA, mycophenolic acid.
