# Supplementary material for: Indications and Long-Term Outcomes of Using Mycophenolate Mofetil Monotherapy in Substitution for Calcineurin Inhibitors in Liver Transplantation
Source: Transpl Int. 2025 Feb 21;38:13790. doi: 10.3389/ti.2025.13790 (PMC11886422; doi:10.3389/ti.2025.13790)
Supplement: Supplementary file 1 [file Table1.DOCX]

**Table 6. (SUPPLEMENTARY)**. Follow-up of diabetes mellitus, hypertension, hematological values, and renal and liver function.

|  | Post-LT  (1mo) | Post-LT (12mo) | Pre-MMF | Pre-MMF-MT  (CNI or MMF-CNI) | MMF-MT  (3-mo) | MMF-MT  (6-mo) | MMF-MT  (12-mo) | MMF-MT (median:68-mo)  (last outpatient review) |
| --- | --- | --- | --- | --- | --- | --- | --- | --- |
| Diabetes mellitus | 123 (38%) | 112 (34.7%) | 114 (35.2%) | 119 (36.7%) | 108 (33.3%) | 105 (32.4%) | 107 (33%) | 105 (32.4%) |
| Hypertension | 126 (39.1%) | 121 (37.5%) | 127 (39.3%) | 128 (39.5%) | 115 (35.5%) | 130 (40.1%) | 130 (40.1%) | 142 (43.8%) |
| Leukocytes/mm^3^ x10^3^ | 5.9 (1.8-136) | 4.9 (1-12) | 5.6 (1.8-15) | 5.5 (2.2-40) | 5.3 (2.3-16) | 5.4 (2-62) | 5.3 (2.1-33) | 5.5 (1.5-87) |
| Hemoglobin (g/dL) | 11 (8-15) | 13.7 (9-16) | 13.4 (8-15.4) | 14 (8-18) | 13.6 (8-18) | 14 (9.3-18) | 14 (8.1-19) | 13.6 (8-18) |
| Platelets/mm^3^ x10^3^ | 158 (11-529) | 129 (34-415) | 142 (35-388) | 160 (34-350) | 165 (14-428) | 164 (37-458) | 165 (35-700) | 167 (17-800) |
| Serum creatinine (mg/dL) | 1.23 (0.4-3.1) | 1.29 (0.5-2.3) | 1.5 (0.5-6.2) | 1.3 (0.6-4.1) | 1.2 (0.4-4.3) | 1.2 (0.5-5.5) | 1.2 (0.5-8) | 1.2 (1-5) |
| GFR (mL/min/1.73m^2^) | 62 (20-134) | 62 (21-122) | 50 (10-126) | 54 (15-126) | 58.3 (14-137) | 59 (10-127) | 58 (7-128) | 60 (7-134) |
| Serum glucose (mg/dL) | 119 (68-363) | 109 (68-300) | 115 (63-400) | 113 (74-381) | 110 (50-294) | 111 (73-277) | 110 (41-350) | 111 (14-400) |
| AST (IU/L) | 21 (5-3504) | 24 (7-512) | 26 (8-353) | 23 (8-252) | 25 (9-306) | 25 (8-554) | 24 (8-281) | 24 (8-322) |
| ALT (IU/L) | 41 (3-3099) | 36 (5-884) | 26 (5-450) | 26 (4-336) | 26 (7-370) | 26 (6-558) | 25 (5-260) | 24 (6-300) |

ALT (alanine amino transferase), AST (aspartate amino transferase); CNI (calcineurin inhibitors); GFR (glomerular filtration *rate);* MMF-MT (mycophenolate mofetil monotherapy)
